# Low-Performing Students Confidently Overpredict Their Grade Performance throughout the Semester

**DOI:** 10.3390/jintelligence11100188

**Published:** 2023-09-28

**Authors:** Meltem Karaca, Lisa Geraci, Nayantara Kurpad, Marcus P. G. Lithander, Steve Balsis

**Affiliations:** 1 Department of Neurology, Boston University Chobanian & Avedisian School of Medicine, Boston, MA 02118, USA; 2 Department of Psychology, University of Massachusetts Lowell, Lowell, MA 01854, USA; lisa_geraci@uml.edu (L.G.); steve_balsis@uml.edu (S.B.); 3 Department of Psychology, St. Mary’s College of Maryland, St. Mary’s City, MD 20686, USA; nkurpad@smcm.edu; 4 Division of Digital Learning, KTH Royal Institute of Technology, 10044 Stockholm, Sweden; mlit@kth.se

**Keywords:** overconfidence, metacognition, miscalibration, predictions

## Abstract

When asked to predict how they will perform on an upcoming exam, students are often poorly calibrated, typically in the direction of overpredicting their performance. Research shows that low-performing students’ calibration tends to remain poor across multiple tests over the course of a semester. We tested whether these students remain confident in these erroneously high grade predictions across the semester or whether their confidence wanes, suggesting some degree of metacognitive awareness. In two studies, students made grade predictions prior to taking four in-class exams and then rated their level of confidence in their predictions. Results from both studies showed that miscalibration and confidence remained stable across tests, suggesting that low-performing students continued to believe that they would perform well on upcoming exams despite prior evidence to the contrary.

## 1. Introduction

Self-awareness is a key measure of intelligence (Ferrari and Sternberg 1998). In the context of a classroom, accurate self-awareness of one’s cognitive abilities and knowledge (metacognition) is associated with high performance (Everson and Tobias 1998; Thiede et al. 2003). Yet, when students are asked to predict their performance on an upcoming exam, they are often poorly calibrated. That is, they either overpredict or underpredict their performance by a significant amount (Hacker et al. 2000). Students who perform the worst on an exam tend to significantly overpredict their performance, often by a few letter grades, whereas students who perform the best on the same exam tend to underpredict performance (Miller and Geraci 2011a). Research shows that students’ calibration often remains poor across multiple tests throughout a semester (e.g., Foster et al. 2017; Tirso et al. 2019). So, the question of interest is: Why do students overpredict their performance in the face of feedback and experience? The goal of the current studies is to attempt to answer this question.

There are a variety of reasons why exam predictions might remain elevated despite the fact that students have test experience and even explicit feedback on their performance (Miller and Geraci 2011a). For example, students may continue to believe that they will perform better than they do because they do not remember or consider their past performance when making predictions (Foster et al. 2017) or because they disregard their past performance and believe that next time will be different, perhaps due to changes in their behaviors or changes in the test material or test difficulty. In both cases, low-performing students, in particular, may continue to overpredict performance and be quite confident in their overpredictions. Alternatively, students may be aware of the fact that they performed more poorly than expected on past exams but may continue to overpredict performance for various reasons. For example, they may decide to make what they consider to be a reasonable guess (perhaps guessing what they think is an average test grade). Or they may base their prediction on what they hope to earn on the exam. In these cases, students may continue to overpredict performance, but their confidence in these overpredictions may be relatively low.

There is good support for the idea that low-performing students over-predict their performance and are not as confident in their predictions compared to high-performing students (Kruger and Dunning 1999; Miller and Geraci 2011b). We also know that students often base their grade predictions, in part, on the grades they hope to achieve (Gramzow et al. 2003; Saenz et al. 2019; Serra and DeMarree 2016). Thus, if students are making their predictions based on wishful thinking, then they may choose to make high grade predictions, but they may not be particularly confident in their predictions, especially after multiple tests with poor performance. On the other hand, if low-performing students are making their predictions based on poor metacognitive information (e.g., Kruger and Dunning 1999), then their confidence in these predictions should be relatively immune to experience and should remain constant even in the face of poor calibration. In other words, if their confidence in their predictions remains unchanged throughout the semester, this pattern of data would suggest that they may be “blissfully unaware” (Kruger and Dunning 1999) and resistant to modifying their predictions.

To examine whether students continue to confidently believe they will perform well on upcoming tests despite contradictory past experiences, we assessed students’ grade predictions across multiple time points and investigated their confidence in these grade predictions (i.e., second-order judgments; SOJs). Second-order judgments have been used before to assess participants’ confidence in their performance predictions and are typically used to measure metacognitive awareness (Dunlosky et al. 2005; Händel and Fritzsche 2013, 2016; Miller and Geraci 2011b). For example, imagine students who are studying for an upcoming exam. At some point prior to the test, the students might assess whether or not they have learned the material. If they are not certain of this assessment, then they might decide to restudy the exam material. The students’ assessments of the accuracy of their metacognitive judgment is considered a second-order judgment and can be useful in regulating a first-order judgment to optimize their learning outcomes (Buratti et al. 2013). According to a two-process hypothesis (Dunlosky et al. 2005), people base their predictions on two processes that cannot be separated: how they think they will perform and their confidence in that assessment. The advantage of using SOJs is that these judgments provide a method for assessing the second process—confidence in one’s prediction. This means that these two judgments are related but not equivalent and that using SOJs can provide useful information beyond what predictions alone provide. For example, SOJs can provide additional information about the basis for the grade predictions that students make. Previously, Miller and Geraci (2011b) showed that low-performing students who overpredicted their exam performance made lower second-order judgments of their exam performance compared to high-performing students (see also Nederhand et al. 2021). Therefore, it is possible that low-performing students have some aspects of metacognitive monitoring, as reflected in their SOJs. Further, it is possible that their SOJs are sensitive to experience and that they could reflect metacognitive learning across the course of a semester, as evidenced by a decrease in SOJs.

In the current studies, we used a methodology similar to that used by Miller and Geraci (2011b) to examine if low-performing students regulate their metacognitive monitoring with the help of feedback from their previous experience. Thus, college students in a psychology course were asked to predict their exam scores over the course of the semester and to rate their confidence in their predictions. The current studies extend previous research by examining not only potential changes in metacognitive judgments using first-order judgments (FOJs) but also potential changes in second-order judgments (SOJs) across a course semester. Only a handful of studies have focused on SOJs in the classroom (e.g., Fritzsche et al. 2018; Miller and Geraci 2011b; Nederhand et al. 2021), and these studies examined students’ SOJs at a single point in time. We assessed confidence in exam predictions over time to provide insight into the basis of students’ overpredictions.

Students in a virtual (Study 1) and an in-person (Study 2) course were asked to make a letter grade prediction immediately before taking each of the four exams throughout the semester. They were asked, “*What letter grade do you think you will receive on this exam?*”. They also rated their level of confidence in their predictions (*Thinking about your grade prediction that you have just made, how confident are you in your prediction of your exam grade?*) using a 5-point Likert scale ranging from 1 to 5. We expected that exam predictions would remain high across tests during the semester and that this would be the case, particularly for the lowest-performing students, replicating prior work (Miller and Geraci 2011b; Tirso et al. 2019). Of interest was what would happen for the second-order judgments (SOJs) for these students. If SOJs decrease over time, this finding would suggest that students’ confidence changes due to their previous experience, though they may hope to perform well in the future (as measured using their exam predictions). If SOJs remain constant or increase over the course of the semester, this finding might indicate that students are not learning from their previous experience and continue to believe that they will perform well on upcoming exams despite their prior performance.

## 2. Study 1

### 2.1. Method

#### 2.1.1. Participants

One hundred ten undergraduate students enrolled in a section of an Introductory to Psychological Science course at a large Northeastern Public University in the United States participated in the study in exchange for course credit. Due to the COVID-19 pandemic, the course was taught in a virtual environment (Spring 2021). The students were largely non-psychology majors. Fifty-nine percent of students at the University of Massachusetts Lowell students are female, 13% Asian, 38% Students of Color, 7% Black/African American, 12% Hispanic/Latino, 58% White, 4% Non-resident Alien, 3% two or more races, and 4% are not specified.

#### 2.1.2. Design and Procedure

At the beginning of the exam, which was administered in Blackboard, participants completed a brief questionnaire in which they were asked to predict the grade they would earn (as a letter grade) and to indicate their level of confidence in their prediction. Note that previous research (Miller and Geraci 2011b) used both letter and number predictions and did not find that this affected calibration, so we chose to examine letter predictions. For the analysis, we converted letter grades to numeric values using the standard grading scale (see Table 1). For example, if participants predicted that they would receive an A-, then we used the numeric value that corresponds to the midpoint of the A- range (i.e., 91). Given that the letter grade prediction question was formed as an open-ended question, some students typed responses that were somewhat outside the standard grading scale. For instance, some students predicted that they would receive a grade between A- and B+. In these cases, we again used the midpoint numeric value of the given range (i.e., the midpoint numeric value for the A- range is 91, and the midpoint numeric value for the B+ range is 88). To calculate the midpoint numeric value, we added 91 and 88 and then divided the total number with 2, which resulted in 89.5). After making a grade prediction, students reported their confidence in their prediction on a 5-item Likert scale that ranged from 1 (low confidence) to 5 (high confidence). All points within the scale were presented with the relevant rating information (1 = not at all confident in my exam prediction, 2 = slightly confident in my exam prediction, 3 = moderately confidence in my exam prediction, 4 = very confident in my exam prediction, 5 = absolutely confident in my exam prediction). Following the rating, students started the exam, which consisted of 30 questions that were administered in the multiple-choice format. The average performance for each exam is as follows (standard deviation is in parenthesis): Exam 1 = 78.55 (10.24), Exam 2 = 80.70 (9.85), Exam 3 = 83.70 (8.98), and Exam 4 = 82.22 (11.01).

### 2.2. Results

The averages for performance, prediction, calibration (difference score) and confidence across four tests are displayed in Table 2^1^.

#### 2.2.1. Calibration and Confidence by Performance Level

Before turning to the main question regarding calibration and confidence across tests, we first examined whether results replicated the common finding that low-performing students overpredict their performance—the “unskilled and unaware” effect (Kruger and Dunning 1999) in the classroom (Miller and Geraci 2011b). To do so, we organized the data into quartiles based on their mean exam performance, with Quartile 1 containing low-performing students and Quartile 4 containing high-performing students. Calibration was calculated by subtracting the exam grade from the predicted score for each participant. Positive values indicated overconfidence, while negative values indicated underconfidence.

Results showed that there was a significant difference in calibration by quartile, *F*(3, 106) = 14.79, *MSE* = 37.02, *p* < .001, η^2^ = 0.30. Bonferroni post hoc tests revealed that students in the top quartile, Quartile 4, were significantly more accurately calibrated (less overconfident) than students in the bottom two quartiles, Quartiles 1 and 2 (*p*s < .05). In addition, students in Quartile 3 were significantly more accurately calibrated than were students in Quartile 1 (*p* < .05). Finally, students in Quartile 2 were significantly more accurately calibrated than were students in Quartile 1 (*p* < .05). These results are consistent with previous findings that low-performing students tend to be relatively miscalibrated compared to high-performing students.

For completeness, we also examined if there was a significant difference in confidence by quartile. Results showed a significant difference in confidence by quartile, *F*(3, 106) = 6.11, *MSE* = 0.26, *p* < .001, η^2^ = 0.15. Bonferroni post hoc tests showed that students in Quartile 1 (low-performing students) were significantly less confident than the students in Quartile 3 (*p* < .05). In addition, students in Quartile 2 were significantly less confident than the students in Quartile 3 (*p* < .05). Although we did not find a significant difference in confidence between the students in the top and bottom quartile, low-performing students (Quartiles 1 and 2) overall were less confident in their exam predictions compared to higher-performing students (i.e., Quartile 3), replicating the general findings from Miller and Geraci (2011b).

#### 2.2.2. Calibration across Tests 

**Figure 1:**
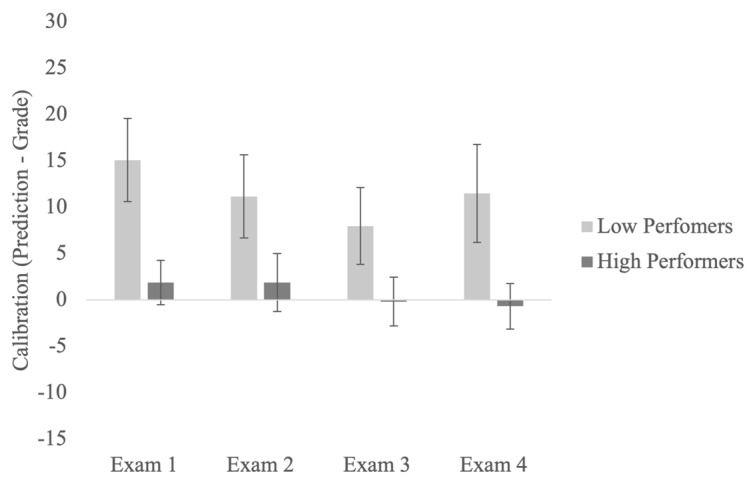
Calibration for the bottom and top quartile participants across four exams.

We investigated low- and high-performing students’ calibration across the semester. To do this, we examined the change in calibration scores across four exams (see Figure 1). Results from a mixed-factor ANOVA suggested that the main effect of the exam was not significant, *F*(3, 153) = 2.64, *MSE* = 74.35, *p* = .05, η_p_^2^ = 0.05. Because the *p* value was at the threshold (given an alpha of 0.05), we performed Bonferroni-corrected pairwise comparisons to examine potential differences in calibration between each exam point. This analysis revealed that all students (regardless of their performance group) were significantly less calibrated at Exam 1 compared to Exam 3 (*p* < .05). Thus, calibration appeared to improve across the first three exams (despite not finding a significant main effect of the exam). The data further showed that the significant change in calibration between Exam 1 and Exam 3 was driven by an increase in performance, as all students performed better on Exam 3 compared to Exam 1 (*p* < .05). The main effect of performance group on calibration was also significant, *F*(1, 51) = 30.99, *MSE* = 194.99, *p* < .001, η_p_^2^ = 0.38. These results replicate previous research showing that low-performing students are more poorly calibrated than high-performing students (Krueger and Mueller 2002; Miller and Geraci 2011b). Finally, the interaction between the exam and performance group was not significant, showing that calibration did not improve over time differentially for low- or high-performing students.

#### 2.2.3. Second-Order Judgments

**Figure 2:**
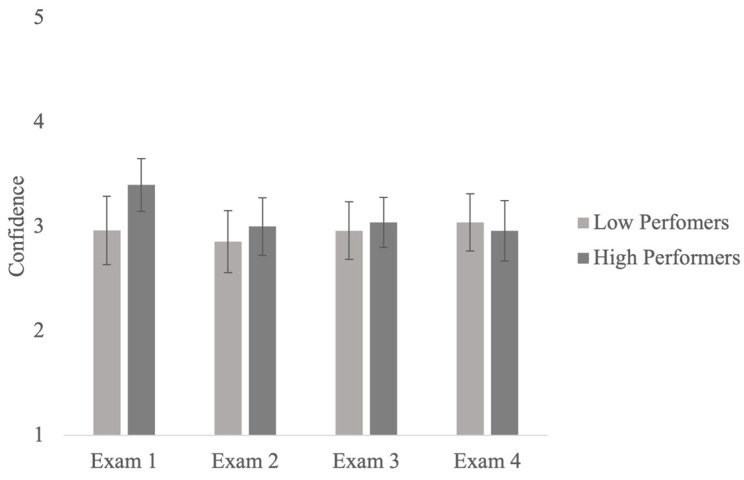
Confidence for the bottom and top quartile participants across four exams.

Turning to the main question of interest: Did confidence in predictions (SOJs) change over the course of the semester? Results from a mixed-factor ANOVA showed that the main effects of the exam, performance group, and their interaction were not significant (see Figure 2). Therefore, the results suggest that low-performing students did not become less confident in their erroneous predictions over the course of a semester. In other words, they did not decrease their confidence after receiving contradictory information (lower exam grades than they predicted).

## 3. Study 2

The goal of Study 2 was to replicate the results of the first study using an in-person sample.

### 3.1. Method

#### 3.1.1. Participants

Sixty-six undergraduate students enrolled in two sections of an Introductory to Psychological Science course at a large Northeastern Public University in the United States participated in the study in exchange for course credit. We used data from two sections (Fall 2021 and Spring 2022) to increase the sample size. Both courses were taught in person by the same instructor using the same grading procedures and course content.

#### 3.1.2. Design and Procedure

The design and procedure were identical to those used in Study 2, with the exception that we presented participants with options for their grade predictions rather than allowing them to write their grade predictions. The reason for this change was to encourage students to enter their responses within the standard grading scale for scoring purposes. Exams again consisted of 30 questions and were multiple-choice. The average performance for each exam was as follows (standard deviation is in parenthesis): Exam 1 = 73.84 (10.77), Exam 2 = 75.05 (12.69), Exam 3 = 76.21 (11.91) and Exam 4 = 77.47 (13.35).

### 3.2. Results

The averages for performance, prediction, calibration (difference score), and confidence across four tests are displayed in Table 3.

#### 3.2.1. Calibration and Confidence by Performance Level

As in Study 1, we investigated whether we obtained the typically “unskilled and unaware” pattern of results. To investigate whether calibration differed across performance levels, we again divided the data into quartiles based on students’ mean exam performance (Quartile 1 = low; Quartile 4 = high). We calculated calibration by subtracting the actual exam score from the predicted score for each participant. Positive values showed overprediction, while negative values showed underprediction.

As expected, results showed a significant difference in calibration by quartile, *F*(3, 62) = 28.69, *MSE* = 35.06, *p* < .001, η^2^ = 0.58. Bonferroni post hoc tests revealed that students in Quartile 4 (high-performing students) were significantly better calibrated (more accurate) than students in Quartiles 1, 2 and 3. In addition, students in Quartile 1 (low-performing students) were relatively miscalibrated compared to students in Quartiles 2 and 3. These findings are consistent with results from Study 1 and with prior research (e.g., Miller and Geraci 2011b; Hacker et al. 2008). For completeness, we again examined if there was a significant difference in confidence by quartile. Results showed that there was no significant difference in confidence by quartile, though the means were in the expected direction.

Given the relatively small numbers of students per quartile, we also examined calibration and confidence by performance level using pooled data from data from both Study 1 and 2 (*N* = 176). Results from one-way ANOVA showed a significant difference in calibration by quartile, *F*(3, 172) = 45.83, *MSE* = 35.99, *p* < .001, η2 = 0.44. Bonferroni post hoc tests showed that students in Quartile 4 (high-performing students) were significantly better calibrated (more accurate) than students in Quartiles 1 and 2. In addition, students in Quartile 3 were significantly better calibrated than students in Quartiles 1 and 2. Lastly, students in Quartile 2 were significantly better calibrated than students in Quartile 1. Thus, overall, low-performing students were relatively miscalibrated compared to high-performing students. These findings are consistent with results reported in previous sections, as well as with prior research (e.g., Miller and Geraci 2011b).

To further examine the unskilled and unaware pattern in our data and the potential downstream effects of initial levels of calibration, we assessed whether there are associations between calibration on one exam and performance on next exam using the pooled data from Studies 1 and 2. Among the entire sample, calibration for Exam 1 was negatively correlated with performance on Exam 2, *r*(174) = −0.34, *p* < .001, 95% CI [−0.46, −0.20], calibration on Exam 2 was negatively correlated with performance on Exam 3, *r*(174) = −0.30, *p* < .001, 95% CI [−0.43, −0.16], and calibration on Exam 3 was negatively correlated with performance on Exam 4, *r*(174) = −0.20, *p* < .01, 95% CI [−0.34, −0.05]. These results indicate that overconfidence decreased as performance increased, which is consistent with the unskilled and unaware pattern (Miller and Geraci 2011b).

For completeness, we again examined if there was a significant difference in confidence by quartile (Miller and Geraci 2011b). Results showed that there was no significant difference in confidence by quartile, though the means were in the expected direction (low-performing students reported numerically lower confidence in their predictions compared to high-performing students).

#### 3.2.2. Calibration across Tests

**Figure 3:**
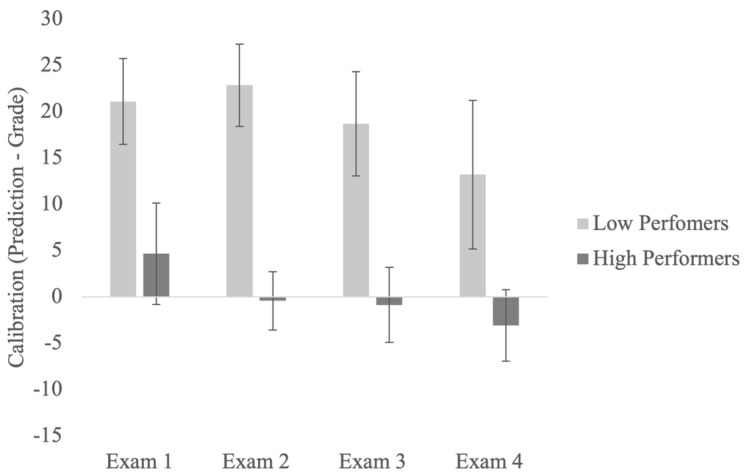
Calibration for the bottom and top quartile participants across four exams.

We assessed students’ metacognitive monitoring over the course of the semester by examining the change in calibration scores across four exams^2^. Results from a mixed-factor ANOVA showed significant main effects of the exam, *F*(3, 90) = 3.75, *MSE* = 98.10, *p* = .014, η_p_^2^ = 0.11. To further examine the significant main effect of the exam, we assessed whether there was a significant difference in calibration between each exam (Exams 1, 2, 3, and 4). Bonferroni-corrected multiple comparisons showed that all students (regardless of their performance group) were more calibrated at Exam 4 compared to Exam 1 (*p* < .05). Thus, calibration appeared to improve across the four exams. Next, we examined if this improvement in calibration was due to decreased predictions, increased performance, or both. Results showed that there was no significant difference in exam performance across four exams. There was also no significant difference in exam predictions across four exams, though the means were in the expected direction such that students overall lowered their predictions from Exam 1 to Exam 4. In addition, there was a significant main effect of performance, *F*(1, 30) = 81.85, *MSE* = 139.43, *p* < .001, η_p_^2^ = 0.73. This finding was consistent with those from Study 1 and previous research (Krueger and Mueller 2002; Miller and Geraci 2011b). Finally, the interaction between exams and performance was not significant (see Figure 3).

To increase power, we assessed students’ metacognitive monitoring over the course of the semester by examining the change in calibration scores across four exams using pooled data from Studies 1 and 2. Results from a mixed-factor ANOVA showed a significant main effect of the exam, *F*(2.65, 219.79) = 4.33, *MSE* = 96.97, *p* = .008, η_p_^2^ = 0.05. Bonferroni-corrected multiple comparisons showed that all students (regardless of their performance group) were more calibrated at Exam 3 compared to Exam 1 and at Exam 3 compared to Exam 2. Thus, pooled data from both studies also indicated that students’ overall calibration improved across the first three exams. Again, we examined if the main effect of the exam could be explained by decreased predictions, increased performance, or both. Results showed that there was no significant difference in exam performance across four exams. However, there was a significant difference in exam predictions across four exams. Bonferroni-corrected multiple comparisons showed that all students (regardless of their performance group) lowered their predictions at Exam 3 compared to 1 and at Exam 4 compared to Exam 1. In addition, there was a significant main effect of performance, *F*(1, 83) = 100.91, *MSE* = 173.83, *p* < .001, η_p_^2^ = 0.55. This finding was consistent with both studies and previous research (Krueger and Mueller 2002; Miller and Geraci 2011b). The interaction between exams and performance was not significant.

#### 3.2.3. Second Order Judgments

**Figure 4:**
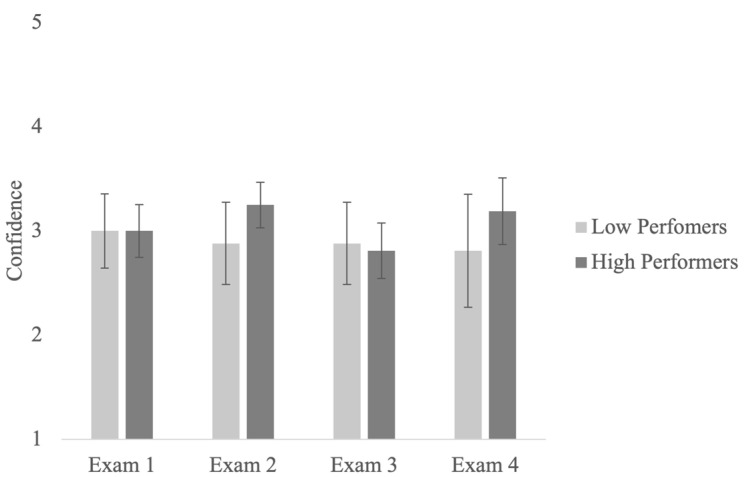
Confidence for the bottom and top quartile participants across four exams.

As in Study 1, the main question of interest was whether students’ confidence in their exam predictions (second-order judgments; SOJs) changed across four exams. Specifically, our goal was to investigate if low-performing students’ SOJs decreased over the course of the semester. Results showed that the main effects of the exam and performance group and their interaction were not significant, showing that students’ SOJs remained the same across four exams (see Figure 4). These findings replicate those from Study 1. Note that the pattern of results remained unchanged when we used the pooled data from both studies.

Because some students can move performance quartiles from one exam to the next, we examined whether the pattern of results held if students were sorted into quartiles based on each exam score rather than on average performance. For this analysis, we used the pooled data from Studies 1 and 2 to increase statistical power. When students were sorted into quartiles based on their Exam 1 and Exam 2 scores, results showed no significant main effects of the exam and performance group and no significant interaction between the exam and performance group. These results are consistent with those reported in Study 1 and Study 2. When students were sorted into quartiles based on their Exam 3 and Exam 4 scores, results showed no significant main effects of the exam and no significant interaction between the exam and performance group. However, there was a significant main effect of the performance group, indicating that high-performing students reported higher confidence compared to low-performing students, *F*(1, 87) = 5.32, *MSE* = 1.42, *p* = .023, η_p_^2^ = 0.06 and, *F*(1, 93) = 5.77, *MSE* = 1.43, *p* = .018, η_p_^2^ = 0.06, respectively. These findings are consistent with prior work, which shows that low-performing students are less subjectively confident in their predictions compared to high-performing students (Miller and Geraci 2011b). 

#### 3.2.4. Relationships across Tests

One might wonder whether students’ predictions and confidence on one test relate to their predictions and confidence on a subsequent test. For example, a student who is highly overconfident on one exam and with high confidence in that prediction might be quite surprised that they underperformed on that exam, and they may report a lower exam prediction on the subsequent exam. Using combined data from Studies 1 and 2 to increase statistical power, we performed a regression model in which the Exam 1 calibration and the Exam 1 SOJs were the independent variables, and the Exam 2 grade prediction was the outcome variable. The results showed that the model was significant, *F*(2, 173) = 18.91, *p* < .001, *R^2^* = 0.18, *R^2^*_adj_ = 0.17. Exam 1 calibration was not associated with the Exam 2 predictions after controlling for Exam 1 confidence (*β* = 0.09, *p* = 0.18). However, Exam 1 confidence was associated with Exam 2 predictions after controlling for Exam 1 calibration (*β* = 0.40, *p* < .001). Thus, reporting higher confidence in Exam 1 predictions was associated with making higher predictions for Exam 2.

We then performed a regression model in which Exam 2 calibration and Exam 2 SOJs were the independent variables, and Exam 3 prediction was the outcome. Results showed that the model was significant, *F*(2, 173) = 7.39, *p* < .001, *R^2^* = 0.08, *R^2^*_adj_ = 0.07. Specifically, the multiple regression analysis showed that Exam 2 calibration was not significantly associated with Exam 3 predictions after controlling for Exam 2 confidence (*β* = 0.03, *p* = .64). However, Exam 2 confidence was significantly associated with Exam 3 predictions after controlling for Exam 2 calibration (*β* = 0.27, *p* < .001). Results show that higher confidence in Exam 2 predictions was associated with higher predictions for Exam 3.

Finally, we performed a regression model in which Exam 3 calibration and Exam 3 SOJs were predictors, and Exam 4 prediction was the outcome. The regression model was statistically significant, *F*(2, 173) = 6.09, *p* < .01, *R^2^*= 0.07, *R^2^*_adj_ = 0.06. Specifically, the multiple regression analysis showed that Exam 3 calibration was not associated with Exam 4 predictions after controlling for Exam 3 confidence (*β* = 0.08, *p* = .27). However, Exam 3 confidence was significantly associated with Exam 4 predictions after controlling for Exam 3 calibration (*β* = 0.23, *p* < .01). Thus, higher confidence in Exam 3 predictions was associated with higher predictions for Exam 4. In sum, across three regression models, we obtained consistent results showing that students are more likely to report higher predictions on subsequent exams if they are confident in their predictions on the current exam.

## 4. General Discussion

Previous research shows that students who perform poorly on exams tend to overestimate their grades compared to their performance, whereas students who perform well on exams do not (Bol and Hacker 2001; Maki and Berry 1984). In addition, low-performing students’ calibration remains poor throughout the course of a semester (Foster et al. 2017). Thus, research shows that low-performing students continue to overpredict their performance even in the face of quite a bit of test experience and sometimes explicit feedback about their calibration (Foster et al. 2017; Miller and Geraci 2011b). We examined whether there was evidence of metacognitive awareness despite these overpredictions. In particular, we tested whether low-performing students might learn from feedback related to their test experience over the course of a semester, as evidenced by lowering their confidence in their predictions.

Results from both studies showed that low-performing students were more miscalibrated compared to high-performing students, replicating prior literature (Miller and Geraci 2011b; Hacker et al. 2000, 2008). In addition, results from both studies showed that calibration remained largely stable across tests for low-performing students, which is consistent with previous research (Foster et al. 2017; Miller and Geraci 2011b). Looking at all students collapsed across performance levels, however, calibration appeared to improve slightly in both studies. In Study 1, students were overall better calibrated in Exam 3 compared to Exam 1 (despite showing no significant main effect of exam). The improvement in calibration may have been due to decreases in students’ exam predictions, increases in their exam performance, or both. The data from Study 1 showed that students performed better on Exam 3 compared to Exam 1, which may explain the improvement in calibration. We should note that the improvement in calibration disappeared in the fourth exam. This may be because the fourth test was a final cumulative exam. In Study 2, students were more calibrated after receiving test experience. Specifically, they showed an improvement in calibration at Exam 4 compared to Exam 1. Further examination of the data showed that students reported numerically lower exam predictions across the semester, although this difference did not reach significance. This pattern of results is slightly different than those in Study 1. It is important to note that the course was taught in person in Study 2, whereas the course was taught virtually in Study 1. Thus, it is possible that students lowered their predictions because the classroom setting fostered greater reflection about their knowledge compared to an online setting. For example, the surrounding conditions such as the presence of peers, might have been helpful in gaining social feedback and assessing learning accordingly (e.g., Bol et al. 2012; Kramarski and Dudai 2009). This is simply speculation, and further research would need to examine how online versus in-person course formats influence metacognitive processes and self-awareness in general. Some may wonder if the observed improvement in calibration indicates regression to the mean rather than real improvements in calibration. It is difficult to determine the contributions of a natural regression to the mean versus other sources of improvement. One counterpoint to the regression-to-the-mean hypothesis is that the high performers also improved, and yet their improved results (at least in Study 2) moved away from the mean and became less accurate (they underpredicted). Additionally, if the regression-to-the mean hypothesis were the best explanation of these data, then one would also expect to see regression to the mean in the SOJ data. However, this result was not obtained. Instead, SOJs remained stable and did not decline.

In this study, we examined what happens to students’ confidence ratings over the course of the semester. Results from both studies showed that second-order judgments remained stable across tests for low- and high-performing students. Prior work suggests that second-order judgments measure individuals’ awareness of their performance prediction accuracy and can assess meta-monitoring (Dunlosky et al. 2005; Washburn et al. 2005). Thus, these findings are consistent with the hypothesis that low-performing students continue to inaccurately monitor their metacognition despite prior evidence to the contrary, thus failing to learn from previous test experiences. Future studies might explore whether providing students with more explicit feedback might influence their second-order judgments (e.g., Tirso et al. 2019). For now, the finding that SOJs remained stable seems to suggest that students are steadfast in their prediction confidence. Further, the differences between predictions and SOJ over time offer additional evidence that these judgments are separable.

The current studies were not designed to directly test specific mechanisms for why students continue to remain confident in their predictions over the course of a semester despite feedback and experience. However, we can speculate about a few possible explanations for why students continued to remain confident in their predictions. One reason might be that they do not remember or use their past performance while making predictions (Foster et al. 2017). It is also possible that they disregard their past performance and determine that the next exam experience will be different due to study or test changes. Or, they may believe that they are better prepared for the exam. Although the design of current studies does not allow us to directly test these various explanations, our results show that low-performing students continue to remain confident in their erroneous exam predictions, which suggests that these predictions may be highly resistant to change.

There are potential limitations to the current studies. For example, in the current study, participants rated their confidence using a scale from 1 (low confidence) to 5 (high confidence), consistent with the prior work (Miller and Geraci 2011b). fResearch shows that the labeling of the scale (using verbal, visual, or various types of numeric points) influences the accuracy of the response (Händel and Fritzsche 2013), so future studies should investigate the influence of the judgment and grading scales when assessing confidence and changes in confidence across the semester. It is possible that using a scale with a greater range would yield greater variability in participants’ responses and show some evidence of learning. Further, it may be helpful to encourage students to use both ends of the scale, because we observed that the distribution of the responses was greatly accumulated around the middle. Thus, future studies may adopt different methods to examine confidence ratings and encourage participants to use the entire scale. Another limitation is that there was no explicit feedback in the current studies (i.e., students could view their exam grades, but they were not told, for example, that their prediction was incorrect). It is possible that seeing one’s exam results served as a type of feedback, though students did not receive any direct feedback about how they performed, and they were not required to access this information, though most did. Research shows that providing students with explicit and concrete feedback may be helpful to show a modest improvement in their metacognitive monitoring (Miller and Geraci 2011a; Experiment 2; but see Foster et al. 2017; Morphew 2021 for different results). Thus, future studies might incorporate a combination of concrete and explicit feedback to examine if there is a change in students’ second-order judgments. Finally, the fact that the tests differed over the course of semester, which is common in a classroom setting, may limit students’ metacognitive learning; however, the results from the current studies are relevant because the presence of different tests mimics the real-world educational settings.

We interpret the lack of change in SOJs across tests for both low- and high-performing students as evidence that there was no metacognitive awareness that resulted from past test or judgment experiences, but we note that we cannot rule out any form of learning. For example, it is possible that people learned from experience but then quickly forgot or disregarded that awareness. For example, Saenz et al. (2019) showed that immediately after feedback, students lower their predictions for their performance on upcoming exams, but their predictions return to being erroneous over time. Additionally, though we interpret SOJs as reflecting metacognitive awareness—an appropriate lack of confidence in one’s prediction—(see also Miller and Geraci 2011b), there’s evidence that SOJs may also reflect other, more stable, attributes of the person making the judgment and not just metacognitive knowledge (Fritzsche et al. 2018). Future research should examine the possible bases of SOJs across different groups of participants and situations.

In summary, the current studies showed that low-performing students’ confidence ratings remained stable over the course of the semester despite the presence of contradictory information (test experience). That is, students who have lower academic grades continued to overpredict future test performance with confidence, suggesting that they believed that they were going to perform well on subsequent exams despite past experience. As metacognitive activities are manifestations of intellectual ability (e.g., Sternberg 1990), these metacognitive biases can hamper low-performing students’ academic performance and distinguish these students from top students (Butler and Winne 1995; Dunlosky et al. 2013). Thus, it is important to design interventions for students to enhance their metacognitive monitoring.

## Figures and Tables

**Table 1:** Grading Scale used in both Studies.

Points	Grade
92.50–100.00	A
89.50–92.49	A−
86.50–89.49	B+
82.50–86.49	B
79.50–82.49	B−
76.50–79.49	C+
72.50–76.49	C
69.50–72.49	C−
66.50–69.49	D+
59.50–66.49	D
00.00–59.49	F

**Table 2:** Mean Prediction, Grade, Calibration, and Confidence (SOJs) for Study 1 by Quartile.

Quartile	Prediction	Grade	Calibration	Confidence
Exam 1				
1 (n = 28)	84.01 (8.29)	68.93 (8.22)	15.08 (12.12)	2.96 (0.88)
2 (n = 31)	87.45 (5.33)	74.52 (5.55)	12.93 (8.31)	3.10 (0.66)
3 (n = 26)	89.96 (6.29)	82.95 (7.44)	7.01 (9.51)	3.35 (0.75)
4 (n = 25)	91.61 (4.66)	89.74 (4.90)	1.87 (6.12)	3.40 (0.65)
Exam 2				
1	82.95 (7.40)	71.79 (9.14)	11.16 (12.14)	2.86 (0.80)
2	85.29 (7.35)	78.61 (7.59)	6.69 (8.96)	2.77 (0.76)
3	88.64 (8.12)	84.36 (6.78)	4.28 (10.87)	3.27 (0.78)
4	91.36 (4.35)	89.47 (5.83)	1.88 (7.96)	3.00 (0.71)
Exam 3				
1	83.68 (7.80)	75.72 (8.55)	7.97 (11.17)	2.96 (0.74)
2	85.75 (6.49)	83.01 (7.86)	2.74 (9.50)	2.74 (0.51)
3	88.29 (5.13)	85.26 (6.20)	3.03 (8.09)	3.50 (0.51)
4	91.70 (5.34)	91.87 (4.42)	−0.18 (6.70)	3.04 (0.61)
Exam 4				
1	81.84 (6.32)	70.36 (12.71)	11.48 (14.22)	3.04 (0.74)
2	85.91 (6.94)	81.72 (5.30)	4.19 (7.64)	2.87 (0.67)
3	87.89 (5.73)	86.54 (6.00)	1.35 (7.20)	3.54 (0.65)
4	90.93 (5.15)	91.60 (4.42)	−0.68 (6.61)	2.96 (0.74)

**Table 3:** Mean Prediction, Grade, Difference, and Confidence (SOJs) for Study 2 by Quartile.

Quartile	Prediction	Grade	Calibration	Confidence
Exam 1				
1 (n = 16)	86.52 (6.55)	65.41 (8.06)	21.10 (9.45)	3.00 (0.73)
2 (n = 17)	87.75 (5.72)	70.59 (7.19)	17.16 (8.76)	2.65 (0.70)
3 (n = 17)	86.71 (5.13)	76.08 (10.15)	10.63 (13.59)	3.00 (0.71)
4 (n = 16)	88.00 (5.32)	83.33 (9.11)	4.67 (11.18)	3.00 (0.52)
Exam 2				
1	83.69 (8.31)	60.83 (8.82)	22.86 (9.07)	2.88 (0.81)
2	84.37 (7.14)	72.35 (11.35)	12.02 (9.49)	3.18 (0.64)
3	85.35 (5.23)	79.41 (6.04)	5.94 (6.42)	3.00 (0.79)
4	87.08 (5.69)	87.50 (5.77)	−0.42 (6.42)	3.25 (0.45)
Exam 3				
1	82.23 (8.75)	63.54 (10.22)	18.70 (11.47)	2.88 (0.81)
2	79.12 (9.11)	76.27 (6.96)	2.85 (11.57)	3.00 (1.23)
3	84.01 (5.55)	77.06 (8.07)	6.96 (10.61)	3.06 (0.90)
4	87.05 (5.40)	87.91 (8.42)	−0.87 (8.29)	2.81 (0.54)
Exam 4				
1	79.23 (15.22)	66.04 (9.98)	13.19 (16.34)	2.81 (1.11)
2	79.97 (7.30)	70.59 (10.49)	9.38 (14.33)	3.06 (0.90)
3	83.74 (7.23)	82.16 (8.16)	1.58 (6.96)	3.00 (1.00)
4	88.17 (3.55)	91.25 (7.97)	−3.08 (7.89)	3.19 (0.66)

## Data Availability

The data presented in this study are available on request from the corresponding author.

## Notes

1 For Exams 2 and 3, three students selected two options while making a judgment related to their confidence. Their first choice was included in the analysis.

2 We pooled the data from both studies and performed the same analysis using the absolute calibration scores. The main finding showing differences between low- and high-performing students remained.
